# The Impact of Single-Dose Alirocumab on Efficacy and Safety After Primary Percutaneous Coronary Intervention in Patients With Acute ST-Segment Elevation Myocardial Infarction: A Single-Center Retrospective Real-World Study

**DOI:** 10.31083/RCM47437

**Published:** 2026-03-12

**Authors:** Pei Wang, Haixia Wang, Dongdong Yan, Zheng Zhang

**Affiliations:** ^1^The First Clinical Medical College, Lanzhou University, 730000 Lanzhou, Gansu, China; ^2^Department of Cardiology, The First Hospital of Lanzhou University, 730000 Lanzhou, Gansu, China; ^3^Gansu Provincial Clinical Research Center for Cardiovascular Diseases, 730000 Lanzhou, Gansu, China

**Keywords:** alirocumab, percutaneous coronary intervention, myocardial infarction

## Abstract

**Background:**

Residual inflammation and persistent lipid abnormalities substantially increase the risk of adverse clinical outcomes in patients with acute myocardial infarction (AMI) undergoing primary percutaneous coronary intervention (PPCI). Although proprotein convertase subtilisin/kexin type 9 (PCSK9) inhibitors have been shown to improve cardiovascular outcomes, the efficacy, safety, and prognosis of these inhibitors when administered as a single dose after PPCI in real-world practice remain unclear.

**Method:**

A retrospective study of patients with acute ST-segment elevation myocardial infarction (STEMI) admitted between May 2023 and May 2024. Patients were assigned to an alirocumab group or a conventional treatment group based on whether a single dose of alirocumab was administered within 6 hours of PPCI. Baseline differences between groups were balanced using 1:1 propensity score matching (PSM). The occurrence of major adverse cardiovascular events (MACEs) at 12 months post-procedure was applied as the primary endpoint. Secondary endpoints included lipid profiles, inflammatory markers, cardiac function, quality-of-life changes, and safety outcomes.

**Results:**

A non-significant downward trend in the incidence of MACEs at 12 months post-PPCI was observed in the alirocumab group compared with the conventional treatment group (log-rank *p* = 0.242). A single dose of alirocumab significantly reduced low-density lipoprotein cholesterol (LDL-C) at 1 month (*p* = 0.011) and attenuated inflammation markers at 24 hours postoperatively. At 12 months, the alirocumab group showed improved cardiac function with significantly reduced left ventricular end-systolic volume (LVESV, *p* = 0.009) and modest but statistically significant improvement in quality of life (*p* = 0.012), primarily driven by enhanced physical activity (*p* < 0.001), alongside reduced insecurity (*p* < 0.001). No increased incidence of adverse events was observed (*p* > 0.05).

**Conclusions:**

This study demonstrated that a single dose of alirocumab in STEMI patients undergoing PPCI was associated with significant improvement in LDL-C levels, attenuation of early postoperative inflammation, and a favorable trend toward improved cardiac function and quality of life, while maintaining an acceptable safety profile.

## 1. Introduction

Acute myocardial infarction (AMI) is a leading cause of death and disability worldwide. The global epidemiologic burden of AMI continues to rise. According to the World Health Organization, the annual incidence of AMI is approximately 50–200 cases per 100,000 people, and approximately 30% of patients face the risk of recurrent cardiovascular events or death within one year of onset [1]. Although primary percutaneous coronary intervention (PPCI) has significantly improved revascularization efficiency among patients with AMI, adverse outcomes including left ventricular remodeling, reinfarction, and heart failure still occur in approximately 15%–20% of patients due to residual inflammation, oxidative stress, and lipid metabolism disorders [2]. Consequently, optimization of perioperative pharmacologic strategies to further improve long-term outcomes has become an important focus of research in cardiovascular medicine.

In recent years, the role of proprotein convertase subtilisin/kexin type 9 (PCSK9) in atherosclerotic cardiovascular disease has garnered substantial attention [3]. Basic research has demonstrated that PCSK9 not only modulates lipid metabolism through promoting low-density lipoprotein receptor (LDLR) degradation but also directly participates in pathological processes, including macrophage activation, plaque destabilization, and cardiomyocyte apoptosis following AMI [4]. PCSK9 inhibitors, represented by evolocumab and alirocumab, have been proven in multiple randomized controlled trials to significantly reduce the incidence of major adverse cardiovascular events (MACE) by effectively lowering low-density lipoprotein cholesterol (LDL-C) levels by approximately 50%–62% and potentially exerting anti-inflammatory effects [5, 6]. The American College of Cardiology/American Heart Association (ACC/AHA) guidelines recommend that patients who have recently experienced acute coronary syndrome (ACS) should first be treated with ezetimibe (Class I recommendation) in addition to intensive statin therapy. If LDL-C levels are still not within the target range, PCSK9 inhibitors may be considered as an additional treatment (Class IIa recommendation) [2].

However, unique clinical challenges exist during the acute phase of ACS, including significant fluctuations in patients’ baseline LDL-C levels caused by stress-induced metabolic changes upon admission, and a delayed maximal lipid-lowering effect of statins, which requires 4–6 weeks of continuous treatment to become apparent. This makes it challenging to accurately evaluate the feasibility of a stepwise lipid-lowering regimen in the early post-PPCI period, and delaying initiation of PCSK9 inhibitors may result in missing the critical window of opportunity to intervene in inflammation and disordered lipid metabolism. Therefore, whether PCSK9 inhibitor therapy should be initiated during the early stages of ACS has become a subject of considerable interest [7]. Several studies published by the European Society of Cardiology (ESC) in 2023 have demonstrated that initiating a combined lipid-lowering strategy involving PCSK9 inhibitors immediately after ACS onset, during hospitalization, or at discharge can rapidly and markedly reduce LDL-C levels, significantly enhancing LDL-C goal achievement rates up to 98% (LDL-C <55 mg/dL), while maintaining a favorable safety profile [8, 9]. However, the routine clinical use of PCSK9 inhibitors is limited by the requirement for injection administration, storage constraints, high cost, availability issues, and dependence on long-term therapy, all of which adversely affect patient adherence. As a result, in clinical practice, many patients receive only a single injection during hospitalization [10]. At the same time, existing clinical evidence has focused primarily on long-term medication regimens, and studies evaluating the single-dose use of PCSK9 inhibitors in the acute phase after PPCI—particularly those based on real-world data—remain scarce. Therefore, the present study was designed to systematically evaluate the efficacy and safety of a single postoperative dose of alirocumab in patients with acute ST-segment elevation myocardial infarction (STEMI) undergoing PPCI, using real-world clinical data and propensity score matching (PSM) to construct comparable cohorts. The aim was to provide additional real-world, evidence-based support for intensive acute-phase post-PPCI intervention strategies and to further clarify the potential value of PCSK9 inhibitors in the acute management of STEMI.

## 2. Materials and Methods

### 2.1 Study Design and Population

This study was designed as a single-center, retrospective, observational cohort study in which clinical data from patients diagnosed with STEMI, admitted to the Cardiology Center of Lanzhou University First Hospital from May 2023 to May 2024, were collected. Throughout the data collection process, the guidelines for strengthening the reporting of observational studies in epidemiology (STROBE) were strictly followed [11]. Patients were assigned to either an alirocumab group (n = 96) or a conventional treatment group (n = 96) based on the administration of a single 75 mg subcutaneous dose of alirocumab within 6 hours following PPCI. This dose was chosen in accordance with the standard clinical starting regimen and pharmacokinetic data showing that a single 75 mg dose produces clinically meaningful LDL-C reductions, while providing a conservative and feasible option for an exploratory single-dose strategy in the acute STEMI setting. Standard guideline-directed medical therapy according to the 2023 ESC guidelines was administered to both groups, including aspirin (loading dose 300 mg, maintenance dose 100 mg/day), ticagrelor (loading dose 180 mg, maintenance dose 90 mg bid), or clopidogrel (loading dose 300 mg, maintenance dose 75 mg/day), and anticoagulant therapy as needed. All patients were initiated on moderate-intensity statin therapy during the index hospitalization (atorvastatin 20 mg/day or rosuvastatin 10 mg/day), and longitudinal prescription records from the electronic medical and pharmacy systems confirmed that all patients in both groups maintained continuous moderate-intensity statin therapy from discharge through 12 months, without ezetimibe co-therapy and without any additional PCSK9 inhibitor use during follow-up. This retrospective cohort study was conducted in accordance with the ethical principles outlined in the Declaration of Helsinki. Ethical approval was obtained from the Ethics Committee of the First Hospital of Lanzhou University, and the requirement for informed consent was waived.

### 2.2 Inclusion and Exclusion Criteria

#### 2.2.1 Inclusion Criteria

Patients aged between 18 and 75 years;

Meets the ESC guidelines for STEMI diagnosis (typical chest pain lasting ≥30 minutes + ST-segment elevation in at least two adjacent leads ≥2 mm (male) or ≥1.5 mm (female) + troponin I ≥ the 99th percentile of the upper limit of normal);

First episode with concomitant PPCI, and post-procedural culprit vessel thrombolysis in myocardial infarction (TIMI) flow grade 3;

Baseline LDL-C ≥1.8 mmol/L.

#### 2.2.2 Exclusion Criteria

Previous use of PCSK9 inhibitors or known allergies to monoclonal antibodies;

Concurrent structural heart disease;

Severe liver dysfunction: alanine aminotransferase (ALT) or aspartate aminotransferase (AST) >120 U/L or renal insufficiency: estimated glomerular filtration rate (eGFR) <30 mL/min/1.73 m^2^;

Patients with active infections, autoimmune diseases, malignant tumors, or psychiatric disorders;

Pregnant or breastfeeding women;

Incomplete clinical data;

Patients who were discharged without authorization.

### 2.3 Definition of Endpoints and Follow-up

#### 2.3.1 Primary Endpoint

The primary endpoint was a composite of MACE at 12 months post-PPCI, including cardiac death (defined as death due to sudden cardiac death, heart failure, or reinfarction), non-fatal reinfarction, unplanned repeat revascularization, malignant arrhythmia, and readmission for heart failure.

#### 2.3.2 Secondary Endpoints

Lipid metabolism markers: LDL-C and total cholesterol (TC) levels at 1, 6, and 12 months post-PCI; Inflammation markers: Neutrophil/lymphocyte ratio (NLR) and C-reactive protein (CRP) at 24 hours post-PPCI; Cardiac function: Cardiac injury markers troponin I (cTNI) and N-terminal pro-B-type natriuretic peptide (NT-proBNP) levels at 6 and 24 hours postoperatively; left ventricular ejection fraction (LVEF), left ventricular end-systolic volume (LVESV), and left ventricular end-diastolic volume (LVEDV) at 6 months and 1 year postoperatively; Health-related quality of life was assessed using the Myocardial Infarction Dimensional Assessment Scale (MIDAS) at hospital discharge (baseline) and at 12 months. At discharge, once patients met clinical stability and discharge criteria, the MIDAS questionnaire was self-completed by patients in a quiet area of the cardiology ward under the supervision of trained cardiology nurses or research staff who were not involved in clinical decision-making. Standardized written and verbal instructions were provided, emphasizing that the items referred to the preceding week, that responses were scored on a 0–4 Likert scale (0 = ‘never’ to 4 = ‘always’), and that there were no right or wrong answers. Staff were available to clarify item wording without suggesting responses, ensuring a uniform administration procedure. Baseline assessments were therefore obtained in clinically stable patients at the time of discharge, reflecting early recovery health status rather than the immediate acute phase. The Scale is a 35-item, myocardial infarction (MI)-specific health status questionnaire with seven domains: physical activity (12 items), insecurity (sense of safety, 9 items), emotional reaction (4 items), dependency (3 items), diet (3 items), concerns about medication (2 items), and side effects (2 items). Each item is rated from 0 (“never” impaired) to 4 (“always” impaired), yielding a total score range of 0–140 points, where higher scores denote worse post-MI health status. This scale has been validated in MI patients, with good internal consistency (Cronbach’s α reported 0.93) and test-retest reliability (Reliability coefficient of retest reported 0.85) [12].

#### 2.3.3 Safety Endpoints

Drug-related adverse events were defined as injection-site reactions (erythema, swelling, persistent pain), upper respiratory tract symptoms (sore throat, runny nose, sneezing), skin itching, hypersensitivity reactions, and muscle pain.

#### 2.3.4 Follow-up Plan

Outpatient follow-up was scheduled at 1, 3, 6, and 12 months after PPCI and included collection of medical history, laboratory testing, and echocardiography. Patients who did not attend outpatient follow-up visits were contacted by telephone to ascertain endpoint events and other prespecified outcomes. As summarized in **Supplementary Fig. 1**, the treatment pathway and dose exposure were identical in both groups except for a single 75 mg alirocumab injection within 6 hours after PPCI in the alirocumab group, on top of continuous moderate-intensity statin therapy without additional lipid-lowering drugs, including ezetimibe or further PCSK9 inhibitor use during the 12-month follow-up.

### 2.4 Statistical Analysis

PSM was performed using a multivariable logistic regression model to estimate propensity scores. A comprehensive set of covariates, all measured before the administration of alirocumab, was included in the model, encompassing demographic characteristics (age, male), clinical characteristics (Killip grade ≥ II, Hypertension, Diabetes, Smoking), laboratory tests (TC, triglyceride (TG), LDL-C, NLR, CRP, cTNI, NT-proBNP, Glucose, glycated hemoglobin (HbA1c), ALT, AST, Creatinine (Cr)), imaging characteristics (LVEF, LVESV, LVEDV), lesion characteristics (Anterior, Posterior, Lateral, Inferior, Multifocal), PCI-related indicators (Preoperative TIMI flow Grade ≤1, Slow blood flow, No-reflow, S2PCI time), and baseline medication status (Ticagrelor, Atorvastatin, Dapagliflozin, RAAS inhibitors, angiotensin receptor-neprilysin inhibitor (ARNI), β-blockers). Patients in the alirocumab and conventional treatment groups were then matched 1:1 using nearest-neighbor matching without replacement, applying a strict caliper width of 0.02 standard deviations of the logit of the propensity score to ensure optimal balance. Balance diagnostics: The balance between matched groups was assessed using standardized mean differences (SMD). After matching, covariates with SMD values <0.1 were considered adequately balanced, indicating negligible residual imbalance. Endpoint analysis: MACE risk was described using Kaplan-Meier curves, and intergroup differences were assessed using the log-rank test and Cox proportional hazards model (adjusted for matching variables); Lipid metabolism indicators, inflammatory markers, cardiac function, quality of life scores, and safety endpoints Intergroup comparisons: Continuous variables were expressed as mean ± standard deviation or median (interquartile range), and intergroup differences were analyzed using independent samples *t*-tests or Mann-Whitney U tests; Categorical variables were expressed as frequencies (%). Differences between groups were assessed using chi-square tests or Fisher’s exact tests. For endpoints measured at multiple time points (TC, LDL-C, cTNI, NT-proBNP, LVEF, LVESV, LVEDV, MIDAS score), between-group comparisons were performed using linear mixed-effects models with random patient intercepts, fixed effects of treatment, time, and treatment × time interaction. Statistical analysis was conducted using SPSS version 26.0 (IBM Corp., Armonk, NY, USA), and a two-sided *p*-value of <0.05 was considered statistically significant.

## 3. Results

### 3.1 Baseline Data of Study Subjects and Intergroup Differences Before and After PSM

**Fig. 1.**
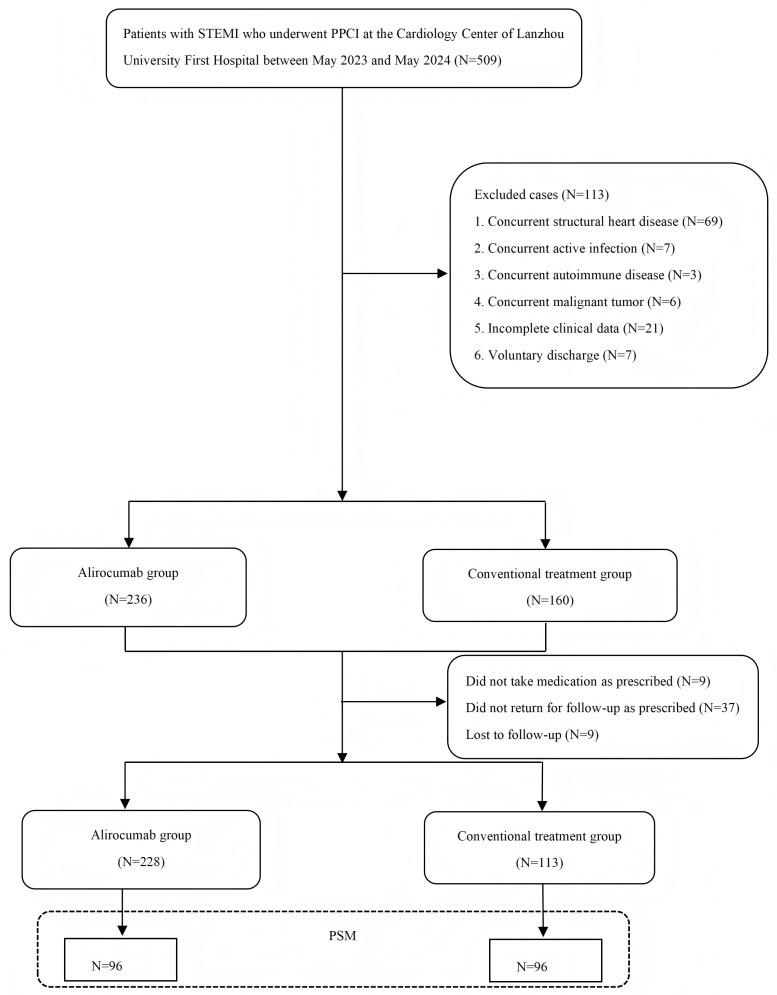
**Flowchart of case screening**. STEMI, ST-segment elevation myocardial infarction; PPCI, primary percutaneous coronary intervention; PSM, propensity score matching.

According to the inclusion and exclusion criteria, 396 patients were initially enrolled, among whom 9 (2.3%) were excluded for nonadherence to discharge medication instructions, 37 (9.3%) were excluded for failure to regularly attend outpatient follow-up visits, and 9 (2.3%) were lost to follow-up (including 5 who lost contact and 4 who declined to participate). After exclusion of these patients, the baseline data of the remaining patients underwent PSM, resulting in a final cohort of 192 matched patients (Fig. 1). Before propensity score matching, there were significant differences between the alirocumab group and the conventional treatment group in terms of age, hypertension, TC, LDL-C, CRP, cTNI, NT-proBNP, LVEDV, and ticagrelor use. After matching, no significant differences were present between the groups for any covariate. The quality of baseline data matching was good (Table 1, **Supplementary Fig. 2**).

**Table 1.** **Baseline characteristics**.

Variable			Before PSM				After PSM			
			Alirocumab (n = 228)	Conventional (n = 113)	SMD	*p* value	Alirocumab (n = 96)	Conventional (n = 96)	SMD	*p* value
Demographic characteristics										
	Age, year		58.3 ± 11.2	61.7 ± 10.5	–0.31	0.007	59.1 ± 10.8	60.3 ± 9.7	–0.09	0.419
	Male, n (%)		164.0 (71.9%)	82.0 (72.6%)	0.01	0.903	70.0 (72.9%)	69.0 (71.9%)	0.02	0.872
Clinical characteristic, n (%)										
	Killip grade ≥ II		35.0 (15.4%)	21.0 (18.6%)	0.09	0.449	16.0 (16.7%)	13.0 (13.5%)	0.09	0.547
	Hypertension		160.0 (70.2%)	57.0 (50.4%)	0.41	<0.001	58.0 (60.4%)	50.0 (52.1%)	0.08	0.246
	Diabetes		82.0 (36.0%)	43.0 (38.1%)	0.04	0.707	21.0 (21.9%)	12.0 (12.5%)	0.08	0.086
	Smoking		115.0 (50.4%)	62.0 (54.9%)	0.09	0.442	58.0 (60.4%)	56.0 (58.3%)	0.04	0.769
Laboratory indexes										
	TC, mmol/L		4.1 ± 0.8	4.7 ± 1.1	–0.64	<0.001	4.0 ± 0.9	4.2 ± 1.2	–0.09	0.193
	TG, mmol/L		1.8 ± 0.6	1.9 ± 0.7	–0.14	0.172	1.7 ± 0.5	1.8 ± 0.6	–0.06	0.264
	LDL-C, mmol/L		2.7 ± 0.6	2.9 ± 0.7	–0.28	0.007	2.7 ± 0.6	2.8 ± 0.8	–0.07	0.328
	NLR		5.1 (3.8–9.5)	5.5 (3.2–11.1)	–0.41	0.402	4.8 (3.9–7.6)	5.4 (3.2–8.9)	–0.08	0.444
	CRP, mg/L		12.5 (2.9–39.5)	8.1 (1.9–20.2)	0.61	0.003	10.0 (2.9–18.5)	12.6 (2.4–19.8)	–0.08	0.531
	cTNI, µg/L		3.2 (0.2–15.0)	1.50 (0.3–6.8)	0.32	0.003	2.5 (0.3–6.1)	1.9 (0.4–5.8)	0.07	0.661
	NT-proBNP, pg/mL		1250.3 (580.2–5930.4)	580.8 (198.1–1600.4)	0.65	<0.001	1290.4 (700.4–1940.4)	1080.3 (630.1–1590.2)	0.09	0.083
	Glucose, mmol/L		7.8 ± 2.1	8.0 ± 2.3	–0.10	0.423	7.7 ± 1.9	7.9 ± 2.0	–0.09	0.478
	HbA1c, %		6.8 ± 1.2	6.9 ± 1.3	–0.09	0.482	6.7 ± 1.1	6.8 ± 1.2	–0.07	0.548
	ALT, U/L		32.4 (18.1–50.4)	35.2 (20.2–55.1)	–0.19	0.241	33.1 (17.4–49.1)	34.2 (19.3–53.4)	–0.07	0.517
	AST, U/L		45.5 (25.6–67.2)	48.3 (28.1–72.2)	–0.21	0.320	46.4 (24.1–66.3)	47.0 (26.3–70.4)	–0.06	0.617
	Cr, µmol/L		78.0 ± 21.0	82.0 ± 24.0	–0.16	0.116	79.0 ± 20.2	80.1 ± 22.3	–0.03	0.742
Imaging characteristics										
	LVEF, %		48.3 ± 8.5	46.9 ± 8.1	0.17	0.147	47.6 ± 6.2	47.8 ± 6.4	–0.02	0.828
	LVESV, mL		65.2 ± 18.1	68.1 ± 20.3	–0.16	0.164	60.0 ± 15.2	56.2 ± 18.4	0.08	0.096
	LVEDV, mL		125.0 ± 25.2	130.4 ± 28.1	–0.24	0.010	117.1 ± 24.1	121.1 ± 26.3	–0.09	0.269
Lesion characteristics										
	Area of infarction, %					0.440				0.860
		Anterior	115.0 (50.4%)	45.0 (39.8%)	0.22		48.0 (50.0%)	46.0 (47.9%)	0.06	
		Posterior	25.0 (11.0%)	15.0 (13.3%)	0.07		10.0 (10.4%)	7.0 (7.3%)	0.07	
		Lateral	28.0 (12.3%)	15.0 (13.3%)	0.03		12.0 (12.5%)	16.0 (16.7%)	–0.09	
		Inferior	42.0 (18.4%)	28.0 (24.8%)	0.16		18.0 (18.8%)	20.0 (20.8%)	–0.08	
		Multifocal	18.0 (7.9%)	10.0 (8.8%)	0.04		8.0 (8.3%)	7.0 (7.3%)	0.04	
PCI-related indicators										
	Preoperative TIMI flow Grade ≤1, %		85.0 (37.3%)	38.0 (33.6%)	0.08	0.510	34.0 (35.4%)	28.0 (29.2%)	0.09	0.356
Post-PCI reflux abnormality										
	Slow blood flow, %		18.0 (7.9%)	10.0 (8.8%)	0.04	0.763	8.0 (8.3%)	5.0 (7.3%)	0.08	0.390
	No-reflow, %		7.0 (3.1%)	5.0 (4.4%)	0.07	0.523	3.0 (3.1%)	3.0 (3.1%)	0.00	>0.999
	S2PCI time, h		6.1 (2.2–15.1)	6.0 (2.3–12.2)	0.16	0.337	6.2 (2.1–15.4)	6.2 (2.4–10.1)	0.09	0.156
Postoperative medication, %										
	Ticagrelor		180.0 (78.9%)	75.0 (66.4%)	0.28	0.012	78.0 (81.3%)	70.0 (72.9%)	0.08	0.171
	Atorvastatin		160.0 (70.2%)	70.0 (61.9%)	0.18	0.128	78.0 (81.3%)	80.0 (83.3%)	–0.06	0.706
	Dapagliflozin		45.0 (19.7%)	18.0 (15.9%)	0.10	0.395	19.0 (19.8%)	18.0 (18.8%)	0.03	0.855
	RAAS inhibitors		90.0 (39.5%)	50.0 (44.2%)	0.10	0.400	38.0 (39.6%)	40.0 (41.7%)	–0.04	0.769
	ARNI		28.0 (12.3%)	12.0 (10.6%)	0.05	0.654	11.0 (11.5%)	6.0 (6.3%)	0.07	0.205
	β-blockers		210.0 (92.1%)	102.0 (90.3%)	0.07	0.567	89.0 (92.7%)	88.0 (91.7%)	0.04	0.789

SMD, standardized mean differences; TG, triglyceride; TC, total cholesterol; LDL-C, low-density lipoprotein cholesterol; NLR, neutrophil/lymphocyte ratio; CRP, c-reactive protein; cTNI, cardiac injury markers troponin I; NT-proBNP, n-terminal pro-B-type natriuretic peptide; HbA1c, glycated hemoglobin; ALT, alanine aminotransferase; AST, aspartate aminotransferase; Cr, Creatinine; LVEF, left ventricular ejection fraction; LVESV, left ventricular end-systolic volume; LVEDV, left ventricular end-diastolic volume; PCI, percutaneous coronary intervention; TIMI, thrombolysis in myocardial infarction; S2PCI, onset to PCI; ARNI, angiotensin receptor-neprilysin inhibitor; RAAS inhibitors, angiotensin receptor blockers or Angiotensin converting enzyme inhibitor.

### 3.2 Efficacy Analysis

#### 3.2.1 Primary Endpoint: MACE Events

**Fig. 2.**
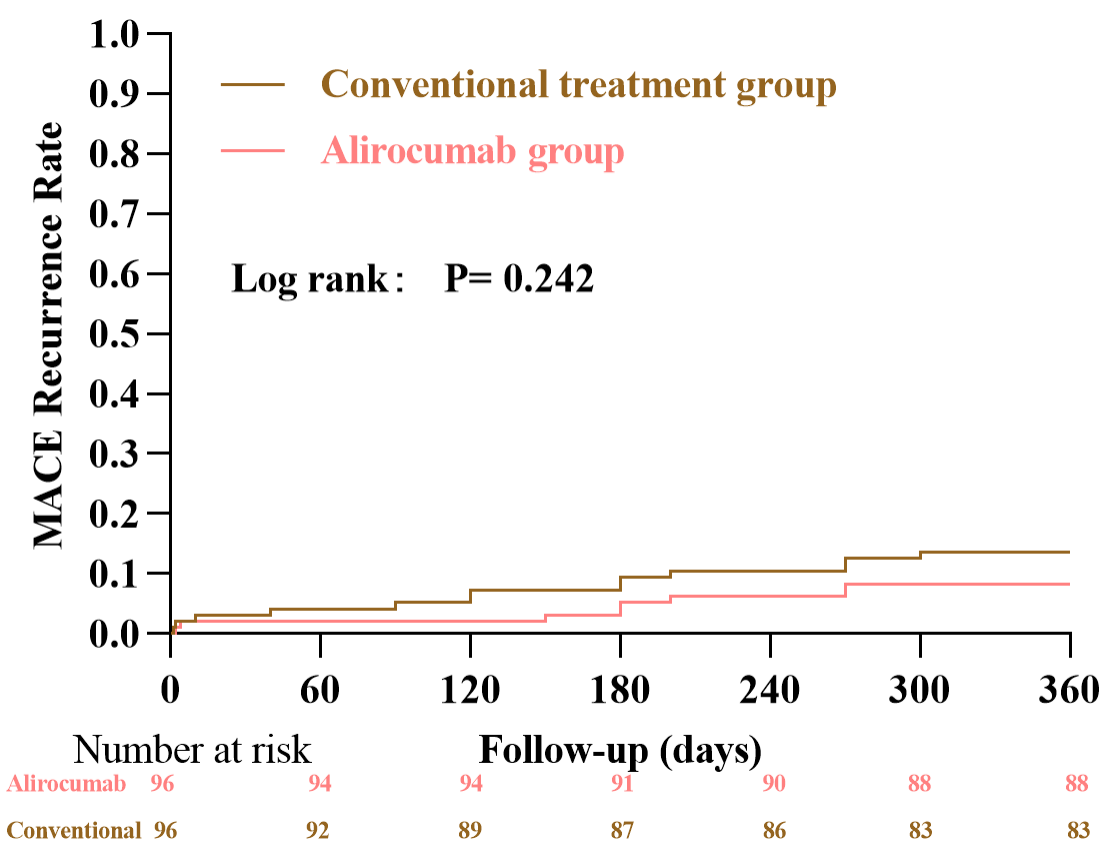
**Cumulative incidence of MACE**. MACE, major adverse cardiovascular events.

During the follow-up period, a total of 21 patients experienced MACE (overall incidence, 10.9%). Kaplan-Meier analysis showed a gradual increase in the cumulative incidence of MACE in both groups, with numerically lower event rates in the alirocumab group compared with the conventional treatment group from approximately day 5 post-PCI; however, this difference did not reach statistical significance (log-rank *p* = 0.242) (Fig. 2). In Cox regression analysis, the incidence of MACE was 8.3% in the alirocumab group and 13.5% in the conventional treatment group, again without a statistically significant between-group difference (*p* = 0.250). The individual components of MACE—cardiac death (2.1% vs. 1.0%), non-fatal myocardial infarction (2.1% vs. 3.1%), unplanned repeat revascularization (2.1% vs. 4.2%), malignant arrhythmia (1.0% vs. 3.1%), and readmission for heart failure (1.0% vs. 2.1%)—also did not differ significantly between the two groups (all *p* > 0.05) (Table 2). Taken together, these findings indicate that, within the limitations of this study, single-dose alirocumab did not demonstrably reduce MACE at 12 months.

**Table 2.** **Comparison of 1-year MACE incidence between the two groups of patients, n (%)**.

Endpoint	Alirocumab group	Conventional treatment group	Adjusted hazard ratio (95% CI)	*p* value
MACE	8.0 (8.3)	13.0 (13.5)	0.606 (0.258–1.423)	0.250
Cardiac death	2.0 (2.1)	1.0 (1.0)	1.973 (0.206–18.947)	0.556
Non-fatal reinfarction	2.0 (2.1)	3.0 (3.1)	0.651 (0.104–4.088)	0.647
Unplanned revascularization	2.0 (2.1)	4.0 (4.2)	0.513 (0.099–2.663)	0.427
Malignant arrhythmia	1.0 (1.0)	3.0 (3.1)	0.319 (0.032–3.148)	0.328
Readmission for heart failure	1.0 (1.0)	2.0 (2.1)	0.486 (0.043–5.467)	0.559

CI, confidence interval.

#### 3.2.2 Secondary Endpoints: Changes in Lipid Metabolism Levels, Inflammation Levels, Cardiac Function Levels, and Quality of Life Scores

In terms of lipid-lowering effects, there were no significant differences in TC levels between the two groups at 1, 6, and 12 months post-PCI (*p* > 0.05). However, the LDL-C levels in the alirocumab group were significantly lower than those in the conventional treatment group at 1 month post-PCI (*p* = 0.011). Consistent with this, a higher proportion of patients in the alirocumab group reached guideline-recommended LDL-C goals at 1 month: more patients achieved LDL-C <1.4 mmol/L, ≥50% reduction from baseline LDL-C, and the combined goal of LDL-C <1.4 mmol/L with ≥50% reduction, compared with the conventional treatment group (**Supplementary Table 1**). The alirocumab group exhibited significantly lower levels of inflammatory markers (CRP and NLR) at 24 hours post-PCI compared with the conventional treatment group (PCRP = 0.040; PNLR = 0.025); additionally, at 1 year postoperatively, LVESV was significantly lower in the alirocumab group than in the conventional treatment group (*p* = 0.009); at 12 months postoperatively, MIDAS scores showed that patients in the alirocumab group had significantly higher quality of life than those in the conventional treatment group at 12 months post-PPCI (*p*= 0.012). Subscale analyses revealed that improvements were predominantly observed in the dimensions of Physical Activity (*p* < 0.001) and Insecurity (*p* < 0.001), both with large effect sizes (Cohen’s d = 0.80 and 0.89, respectively). Other dimensions, including Emotional Reaction, Dependency, Diet, Medication Concerns, and Medication Side-effects, showed no significant differences between groups (*p* > 0.05) (Table 3).

**Table 3.** **Comparison of lipid metabolism levels, inflammation levels, cardiac function levels, and quality of life scores between the two groups during follow-up**.

Variable			Alirocumab group	Conventional treatment group	*p* value
TC, mmol/L					
	1 month		3.2 ± 1.2	3.5 ± 1.1	0.066
	6 months		3.1 ± 0.9	3.3 ± 1.1	0.251
	12 months		3.1 ± 0.9	3.2 ± 1.2	0.432
LDL-C, mmol/L					
	1 month		1.9 ± 0.6	2.2 ± 0.5	0.011
	6 months		2.2 ± 0.5	2.4 ± 0.7	0.043
	12 months		2.3 ± 0.6	2.4 ± 0.6	0.196
CRP, mg/L					
	24 hours		3.5 (3.0–7.9)	6.3 (1.9–11.0)	0.040
NLR					
	24 hours		3.2 ± 2.2	3.9 ± 2.1	0.025
cTNI, µg/L					
	6 hours		4.8 (2.2–16.1)	5.50 (3.6–13.0)	0.541
	24 hours		1.5 (0.2–5.2)	2.20 (1.2–6.5)	0.129
NT-pro BNP, pg/mL					
	6 hours		580.2 (329.8–1089.8)	650.2 (219.5–1279.8)	0.463
	24 hours		349.8 (230.1–680.3)	420.3 (329.6–799.8)	0.358
LVEF, %					
	6 months		48.6 ± 5.8	49.9 ± 6.2	0.255
	12 months		47.8 ± 6.3	48.6 ± 5.5	0.512
LVESV, mL					
	6 months		46.5 ± 16.8	50.2 ± 15.7	0.067
	12 months		45.2 ± 15.3	49.6 ± 12.1	0.009
LVEDV, mL					
	6 months		109.2 ± 25.3	115.5 ± 23.8	0.157
	12 months		98.1 ± 22.8	109.3 ± 28.2	0.212
MIDAS score					
	Before discharge (Total score)		75.6 ± 10.1	78.3 ± 9.5	0.213
		Physical Activity	29.1 ± 5.5	27.9 ± 4.9	0.356
		Diet	2.8 ± 1.0	3.0 ± 1.1	0.512
		Emotional Reaction	8.2 ± 2.3	8.9 ± 1.9	0.426
		Dependency	5.9 ± 2.1	5.8 ± 1.8	0.351
		Security	19.9 ± 5.0	21.5 ± 4.6	0.167
		Medication Concerns	5.2 ± 1.8	5.6 ± 1.6	0.138
		Medication Side-effects	4.5 ± 1.0	5.6 ± 1.7	0.239
	12 months (Total score)		36.6 ± 7.1	38.9 ± 8.2	0.012
		Physical Activity	12.8 ± 3.5	14.8 ± 4.0	<0.001
		Diet	2.6 ± 0.9	1.9 ± 1.0	0.107
		Emotional Reaction	4.2 ± 1.3	3.4 ± 1.4	0.246
		Dependency	2.9 ± 1.1	2.1 ± 1.2	0.430
		Security	9.5 ± 2.6	12.0 ± 3.0	<0.001
		Medication Concerns	2.2 ± 0.8	2.3 ± 0.9	0.532
		Medication Side-effects	2.4 ± 0.9	2.4 ± 1.0	0.769

MIDAS, Myocardial Infarction Dimensional Assessment Scale.

### 3.3 Safety Analysis

During the follow-up period, 7 adverse events were recorded: 5 (5.2%) in the alirocumab group and 2 (2.1%) in the conventional treatment group: persistent pain at the injection site (2.1% in the alirocumab group vs. 0% in the control group, *p* = 0.156), sore throat (2.1% in the alirocumab group vs. 0% in the control group, *p* = 0.156), skin itching (1.0% in the alirocumab group vs. 0% in the control group, *p* = 0.317), muscle pain (alirocumab group 0% vs. control group 2.1%, *p* = 0.156). Overall, administration of a single dose of alirocumab was not associated with an increased incidence of adverse reactions (alirocumab group: 5.2% vs. control group: 2.1%, *p* = 0.249) (Table 4).

**Table 4.** **Comparison of adverse reactions between the two groups, n (%)**.

Variable		Alirocumab group	Conventional treatment group	*p* value
Injection site reactions				
	Erythema	0	0	>0.999
	Swelling	0	0	>0.999
	Persistent pain	2 (2.1)	0	0.156
Upper respiratory tract symptoms				
	Sore throat	2 (2.1)	0	0.156
	Runny nose	0	0	>0.999
	Sneezing	0	0	>0.999
Skin itching		1 (1.0)	0	0.317
Hypersensitivity reactions		0	0	>0.999
Muscle pain		0	2 (2.1)	0.156
Total		5 (5.2)	2 (2.1)	0.249

## 4. Discussion

In recent years, the importance of early intensive lipid-lowering therapy in ACS has been increasingly emphasized by international guidelines. According to cholesterol management guidelines in Europe, the United States, and China, high-intensity statin therapy should be initiated at the time of hospital admission for patients with ACS, regardless of baseline LDL-C levels. For patients at extremely high risk whose LDL levels remain above target, other lipid-lowering drugs such as ezetimibe should be added as soon as possible, and PCSK9 inhibitors should be introduced when necessary to rapidly reduce LDL-C to guideline-recommended target levels [2, 13]. However, in real-world clinical practice, rates of lipid-lowering therapy intensification and LDL-C target achievement among patients with ACS remain generally low, providing a rationale for earlier initiation of PCSK9 inhibitor therapy [14]. Several clinical studies, including EVACS [15], EVOPACS [8] and EPIC-STEMI [9], have demonstrated that initiating PCSK9 inhibitor therapy at admission significantly reduces LDL-C levels within days following ACS onset, enabling patients to achieve lipid targets rapidly. A meta-analysis of nine studies involving 2869 ACS patients confirmed that early initiation of PCSK9 inhibitor therapy in the hospital setting can rapidly and significantly lower LDL-C levels, improve lipid control rates, and significantly reduce the risk of MACE in the short term, with good safety [16]. These studies suggest that initiating PCSK9 inhibitor-enhanced lipid-lowering therapy in the early stages of ACS onset is a promising strategy. However, in actual clinical application, factors such as the injection route, storage requirements, high cost, and compliance challenges associated with long-term therapy have resulted in low patient adherence and high discontinuation rates. In routine clinical practice, the use of PCSK9 inhibitors is frequently limited to single in-hospital injections [10]. Previous studies have primarily evaluated long-term, multiple-dose regimens, while the short-term and long-term impacts of single-dose administration have rarely been assessed. The present study addresses this gap by evaluating, for the first time, the efficacy and safety of a single dose of alirocumab in patients with STEMI undergoing primary PCI in a real-world clinical setting.

In this real-world, propensity score–matched cohort, the cumulative incidence of MACE over 12 months was numerically lower in the alirocumab group than in the conventional treatment group; however, this difference was not statistically significant in either Kaplan-Meier or Cox regression analyses, and the individual components of the composite endpoint (cardiac death, non-fatal myocardial infarction, unplanned revascularization, malignant arrhythmia, and rehospitalization for heart failure) were also similar between groups. Given the modest sample size (n = 192) and low absolute event count (n = 21), the study was likely underpowered to detect anything other than large treatment effects, as the statistical power of time-to-event analyses is strongly driven by the number of events. Consequently, the observed numerical differences should be regarded as exploratory and hypothesis-generating rather than as evidence of a definite clinical benefit of single-dose alirocumab. Our neutral primary endpoint contrasts with the robust risk reductions reported in large randomized trials of long-term PCSK9 inhibition, such as FOURIER (evolocumab) and ODYSSEY OUTCOMES (alirocumab), in which sustained LDL-C lowering over several years led to significant reductions in MACE. Thus, while our findings are directionally consistent with the pharmacologic effects of PCSK9 inhibition, they do not demonstrate a clinical efficacy signal for a single peri-procedural dose and instead underscore the need for adequately powered, multicenter studies with longer follow-up to clarify whether short-term or peri-PCI PCSK9 inhibition can meaningfully influence prognosis after STEMI.

Although no significant difference in the primary endpoint of MACE was observed, superiority of the alirocumab group was evident across several secondary endpoints: (1) In terms of lipid levels, the LDL-C levels in the alirocumab group were significantly lower than those in the conventional treatment group at 1 month post-PPCI. Nevertheless, the mean LDL-C values observed in the alirocumab group (1.9 ± 0.6 mmol/L at 1 month) remained numerically higher than the more stringent LDL-C goal of <1.4 mmol/L (<55 mg/dL) proposed for many very high-risk patients in recent ESC/EAS and ACC documents. These findings are consistent with real-world data showing that achieving such intensive LDL-C levels can be challenging in routine clinical practice, even among very high-risk patients. In our cohort, all patients received moderate-intensity statin therapy, and ezetimibe was not prescribed, reflecting local treatment patterns during the study period rather than a protocol-mandated exclusion. From a pharmacokinetic standpoint, a single 75 mg subcutaneous injection of alirocumab reaches peak concentrations approximately 5–7 days after administration, has a half-life of about 17–20 days, and can maintain LDL-C lowering for more than 4–8 weeks, which likely contributed to the early reductions in LDL-C observed at 1 month [17]. Although our study demonstrated significant reductions in LDL-C levels in the alirocumab group at 1 month post-PPCI, this did not translate into a statistically significant reduction in MACE at 12 months. In this context, our findings may reflect a therapeutic gap: although early reductions in LDL-C may contribute to short-term plaque stabilization, the lack of sustained PCSK9 inhibition likely limits long-term event reduction. Moreover, when comparing our LDL-C results with current clinical targets, the limitations become clearer. According to the 2023 ESC and ACC guidelines, post-ACS patients are advised to achieve LDL-C levels below 1.4 mmol/L. In our cohort, the alirocumab group achieved a mean LDL-C of 1.9 ± 0.6 mmol/L at 1 month—significantly lower than the conventional treatment group, but still above the recommended threshold. These suboptimal target attainments, together with the limited duration of PCSK9 inhibition, may explain why no significant difference in MACE was observed in this real-world study. (2) With respect to inflammatory markers, CRP and NLR at 24 hours after PPCI were significantly lower in the alirocumab group than in the conventional treatment group, suggesting that alirocumab may exert anti-inflammatory effects in the acute phase of STEMI, consistent with the anti-inflammatory effects of PCSK9 inhibitors reported in previous basic and clinical studies [18]. However, previous meta-analyses have indicated that PCSK9 inhibitors have no significant effect on systemic inflammatory markers such as serum hs-CRP [19]. This discrepancy may be attributed to differences in the timing of biomarker assessment; prior studies (e.g., FOURIER, ODYSSEY) measured CRP several weeks post-PCI, whereas this study captured inflammatory changes within 24 hours of PPCI—a period marked by high inflammatory activity—suggesting a potential acute anti-inflammatory effect of alirocumab. (3) In terms of cardiac function, LVESV in the alirocumab group was significantly lower than that in the conventional treatment group at 1 year after PPCI, indicating a milder degree of ventricular remodeling after myocardial infarction in the alirocumab group. This reduction is clinically relevant, as LVESV is a sensitive and well-established surrogate for left ventricular remodeling following myocardial infarction. Importantly, a decline in LVESV often reflects the occurrence of reverse remodeling (RR)—a therapeutically driven, favorable change in cardiac geometry and function that rarely occurs spontaneously and is considered the structural foundation for improved symptoms and prognosis across cardiovascular disease treatments [20, 21, 22]. The significant reduction in LVESV in the alirocumab group may therefore indicate that early PCSK9 inhibition not only improved lipid and inflammatory profiles, but also promoted favorable structural remodeling. This aligns with prior studies such as Ziogos *et al*. [23], which reported that early administration of evolocumab reduced myocardial inflammation and mitigated the increase in LVESV. Taken together, these findings support a mechanistic sequence whereby acute anti-inflammatory action limits early myocardial injury and facilitates subsequent RR. Additionally, this structural benefit was accompanied by a significantly greater improvement in patient-reported quality of life (MIDAS score) at 12 months in the alirocumab group. While LVEF remained unchanged, the observed LVESV reduction and associated improvement in functional outcomes support the notion that reverse remodeling had occurred and was clinically meaningful. Therefore, although LVESV was the only cardiac parameter with significant between-group differences, it represents a key structural marker of therapeutic benefit and potentially favorable long-term cardiac outcomes. (4) In terms of quality of life, at 12 months post-PPCI, the total MIDAS score in the alirocumab group was significantly lower compared to the conventional treatment group (36.6 ± 7.1 vs. 38.9 ± 8.2, *p* = 0.012). Although statistically significant, the absolute difference (≈2.3 points) is modest relative to conventional thresholds for minimal clinically important difference (MCID), which typically range from approximately 5% to 10% of the scale for cardiovascular-specific quality-of-life measures. This suggests that the observed difference, while meaningful statistically, might not fully reach clinical perceptibility at the total score level. However, the subscale analysis revealed clinically meaningful differences within specific domains. The alirocumab group showed significant improvements in Physical Activity (12.8 ± 3.5 vs. 14.8 ± 4.0, *p* < 0.001; Cohen’s d ≈ 0.80) and Security (9.5 ± 2.6 vs. 12.0 ± 3.0, *p* < 0.001; Cohen’s d = 0.89), reflecting substantial gains in daily physical functioning and psychological confidence regarding disease recurrence. These findings are consistent with with previous studies demonstrating improvements in mobility, reductions in anxiety, and overall well-being associated with PCSK9 inhibitors therapy [24, 25]. Other MIDAS dimensions such as Emotional Reaction, Dependency, Diet, Medication Concerns, and Side-effects did not show significant between-group differences, suggesting the quality-of-life benefits from alirocumab are primarily concentrated in improved physical performance and reduced health-related anxiety. Taken together, these subscale findings clarify that the clinical value of alirocumab in improving post-MI quality of life is most pronounced in enhancing physical capability and providing emotional reassurance, even if the between-group difference in the total MIDAS score remains modest.

In addition, the safety analysis showed that the incidence of adverse reactions was low in both groups and that the between-group difference was not statistically significant. Administration of a single dose of alirocumab was not associated with a significant increase in the risk of adverse events. Most adverse events were mild, including injection-site pain and throat discomfort, and all resolved spontaneously within 3 days. This finding is consistent with the safety profile observed after a single dose of alirocumab in healthy subjects.

### Limitations

Several limitations should also be acknowledged in this study: (1) The single-center, retrospective, observational design may have introduced selection and information bias. Moreover, as with all observational ‘real-world’ analyses, although PSM was employed to balance known baseline confounders, unmeasured or residual biases may still have influenced outcome comparability, thus limiting the ability to draw conclusions with the same level of certainty as randomized clinical trials. Differences in patient demographics, healthcare systems, procedural techniques, and background medical therapy across regions and centers may limit the external validity of our results. (2) The modest overall sample size and the limited number of primary endpoint events not only reduced the statistical power of the study but also constrained the performance of the propensity score matching, thereby increasing the uncertainty around the estimated treatment effects. Additionally, the follow-up duration was limited to 1 year, which may have been insufficient to capture long-term clinical benefits. For atherosclerotic cardiovascular disease, the long-term benefits of many interventions often become apparent only after longer-term follow-up. (3) As a retrospective analysis, data collection was limited to predefined follow-up protocols, restricting the availability of additional clinical indicators and thereby weakening the strength of the conclusions. (4) Potential discrepancies between patient recall and actual clinical events may have introduced recall bias. Adherence to statin therapy was assessed indirectly based on longitudinal prescription and refill records rather than objective measures such as pill counts or electronic monitoring. Although these data suggest that all patients remained on moderate-intensity statins during follow-up, this approach cannot fully capture actual medication intake, and residual misclassification of adherence cannot be excluded. (5) CRP and NLR are dynamic inflammatory markers that can be influenced not only by the acute ischemic event itself but also by multiple peri-procedural factors, including contrast load, procedural complexity, periprocedural myocardial injury, perioperative infections, hemodynamic instability, and concomitant medications such as heparins, statins, and β-blockers. Previous studies have shown that CRP and NLR after PCI or MI are strongly affected by the overall inflammatory burden and procedural stress rather than a single therapeutic intervention alone, which may confound the observed between-group differences. Thus, the reduction in CRP and NLR at 24 hours should be interpreted with caution and considered exploratory. (6) Although no significant difference in the incidence of adverse events was observed between the alirocumab and conventional treatment groups and most events were mild and self-limited, the absolute number of adverse events in both groups was very low. As a result, the study has limited statistical power to detect infrequent or rare safety signals, and our findings cannot be interpreted as definitive evidence of safety in the broader STEMI population. Our results should instead be viewed as exploratory and consistent with the established safety profile of alirocumab reported in large phase III programs, rather than as a comprehensive safety evaluation. Larger prospective studies with longer follow-up are required to more robustly characterize the safety of alirocumab in the acute post-PPCI setting. Given these limitations, our findings should be interpreted as hypothesis-generating and warrant confirmation in larger, prospective, multicenter cohorts or randomized trials.

## 5. Conclusions

In summary, this study suggests that, in patients with STEMI undergoing PPCI, postoperative administration of a single dose of alirocumab may provide more effective LDL-C lowering, attenuation of early inflammatory responses, improvement in ventricular remodeling, and enhancement of quality of life than conventional therapy, while maintaining a favorable safety profile. Overall, these real-world findings support the potential clinical value of administering a single dose of alirocumab after PPCI in patients with STEMI.

## Availability of Data and Materials

The datasets used and analysed during the current study are available from the corresponding author on reasonable request.

## Supplementary Material

Supplementary material associated with this article can be found, in the online version, at https://doi.org/10.31083/RCM47437.
