# Supplementary material for: The Impact of Single-Dose Alirocumab on Efficacy and Safety After Primary Percutaneous Coronary Intervention in Patients With Acute ST-Segment Elevation Myocardial Infarction: A Single-Center Retrospective Real-World Study
Source: Rev Cardiovasc Med. 2026 Mar 12;27(3):47437. doi: 10.31083/RCM47437 (PMC13036518; doi:10.31083/RCM47437)
Supplement: Supplementary file 1 [file 2153-8174-27-3-47437-s1.zip › STROBE checklist.pdf]

STROBE Statement: Checklist of Essential Items for Observational Studies

**Author Information**			
Journal to send: RCM	First author:Pei Wang	Corresponding author:Zheng Zhang	
Manuscript ID:RCM47437	Article title: **The Impact of Single-Dose Alirocumab on Efficacy and Safety After Primary Percutaneous Coronary Intervention in Patients With Acute ST-Segment Elevation Myocardial Infarction: A Single-Center Retrospective Real-World Study**		

**Content and Topic**	**Entry number**	**Check items (**tick if applicable)	**Location**
**Title and Abstract**	1	☑The title or abstract should include commonly used professional terminology to describe the research design.<br>☑The abstract should be comprehensive and accurately and fluently describe what was done and what was found in the study.	p.1
**Preface**			
Background or Principle	2	☑ Explain the reported research background and rationale	pp.1–2
Target	3	☑ Elucidate the research objectives, including any predefined hypotheses	p.2
**Method**			
Research Design	4	☑ The elements of the research design were presented earlier in the paper.	p.2
Research Site	5	☑Describe the study site, specific location, and relevant time frame (including the periods for participant recruitment, exposure, follow-up, and data collection).	p.2
Subject investigated	6	☑Cohort study: describe the eligibility criteria, source population, and selection methods for study subjects, as well as the follow-up methods.<br>□Case-control study: describe the eligibility criteria, source population, and selection methods for identifying cases and controls, as well as the rationale for selecting cases and controls;<br>□Cross-sectional study: Describes the eligibility criteria, source population, and selection methods for the study subjects.<br>□Cohort study-paired study: describe the criteria for pairing and the number of exposed and non-exposed individuals;<br>□Case-control study-matched study: Describe the matching criteria and the number of controls corresponding to each case	pp.2–3
Research Variables	7	☑Clearly define the outcome, exposures, predictors, potential confounders, and effect modifiers (provide diagnostic criteria if possible).	pp.2–3
Data Source or Measurement	8*	☑For each variable of interest, describe its data source and detailed determination (measurement) methods (if multiple groups are involved, also describe the comparability of the determination methods between groups).	pp.2–3
Bias	9	☑ Process of describing and explaining potential biases	pp.11–12
Sample Size	10	☑ Explanation of the method for determining sample size	pp.4–5
Quantitative Variable	11	☑ Explain how to handle measurement variables in the analysis (if possible, describe how to select strata and the rationale for strata selection)	p.4
Statistical Methods	12	☑Describe all statistical methods, including those for controlling for confounding;<br>□Describe the subgroup and interaction testing methods;<br>□Describe the missing value handling method;	pp.3–4
		☑Cohort study: if possible, explain the handling of lost-to-follow-up cases;<br>Case-control study: if possible, describe the matching method for cases and controls;<br>Cross-sectional studies: if possible, describe the statistical methods determined according to the sampling strategy□Describe sensitivity analysis	pp.3–4
**Bear fruit**			
Subject Investigated	13*	☑The number of subjects at each stage of the study, such as the number that may have qualified, the number that were tested for qualification, the number that were confirmed qualified, the number included in the study, the number that completed follow-up, and the number that were analyzed;<br>☑Describe the reasons for the failure of the subjects to participate in each phase of the study;<br>☑Consider using a flowch- art	pp.4–5
Descriptive Data	14*	☑Describe the characteristics of the study subjects (e.g. demographic, clinical and social characteristics) and information on exposures and potential confounders;<br>□The number of subjects with missing values for each variable of interest;<br>☑Cohort study: Summarize follow-up time (e.g., mean time and total time)	pp.6–7

Outcomes Data	15*	☑cohort study: reporting the number of outcome events that occur or the number of outcome events that occur according to time;<br>□case-control study: reporting the number of exposures or composite measures of exposure for each category;<br>□Cross-sectional study: Number of reported outcome events or summary of measured exposures	pp.8–10
Main Results	16	☑Provide estimates of association strength and precision (e.g. 95%*CI*) for unadjusted and adjusted for confounding factors, and specify which confounding factors were adjusted for and the rationale for their selection;<br>☑When grouping continuous variables, report the group boundaries;<br>☑If applicable, the relative risk during the meaningful period can be converted to an absolute risk.	pp.8–10
Other Analyses	17	□Other analyses conducted in the report, such as subgroup and interaction analyses, as well as sensitivity analyses	N/A
**Discussion**			
Important Results	18	☑Key findings related to the research hypothesis	pp.8–12
Boundedness	19	☑Discuss the limitations of the study by considering potential biases and imprecise sources; discuss the direction and magnitude of potential biases.	pp.11–12
Explain	20	☑A comprehensive interpretation of the overall findings should be cautiously presented, taking into account the study objectives, limitations, multivariate analysis, results of similar studies, and other relevant evidence.	pp.8–12
Generalizability	21	☑Discuss the generalizability of the research findings (validity of extrapolation)	p.12
**Other Information**			
Aid financially	22	☑Specify the funding sources and sponsors of the current study (if possible, provide the funding details for the original research)	p.12

*Provide case and control information in case-control studies; if possible, provide exposure and non-exposure group information in cohort and cross-sectional studies.
