# Supplementary material for: The Impact of Single-Dose Alirocumab on Efficacy and Safety After Primary Percutaneous Coronary Intervention in Patients With Acute ST-Segment Elevation Myocardial Infarction: A Single-Center Retrospective Real-World Study
Source: Rev Cardiovasc Med. 2026 Mar 12;27(3):47437. doi: 10.31083/RCM47437 (PMC13036518; doi:10.31083/RCM47437)
Supplement: Supplementary file 1 [file 2153-8174-27-3-47437-s1.zip › supplementary figures-captions.docx]

Supplementary Fig. 1 Legend: The schematic illustrates the study design, interventions, and assessment timepoints for propensity score-matched patients receiving either a single dose of alirocumab plus standard therapy (Alirocumab Group, n=96) or standard therapy alone (Conventional Treatment Group, n=96) following primary PCI. Key timepoints include baseline (PPCI), the 6-hour alirocumab administration window, and follow-up assessments at 24 hours, 1, 6, and 12 months. Standard guideline-directed medical therapy, including dual antiplatelet therapy and moderate-intensity statins, was maintained in both groups throughout the study. Abbreviations: LDL-C, low-density lipoprotein cholesterol; TC, total cholesterol; NLR, neutrophil-to-lymphocyte ratio; CRP, C-reactive protein; cTNI, cardiac troponin I; NT-proBNP, N-terminal pro-B-type natriuretic peptide; LVEF, left ventricular ejection fraction; LVESV, left ventricular end-systolic volume; LVEDV, left ventricular end-diastolic volume; MIDAS, Myocardial Infarction Dimensional Assessment Scale; MACE, major adverse cardiovascular events.

Supplementary Fig. 2 Legend: Each point represents the absolute standardized mean difference for a baseline covariate between the treatment and control groups. Red and blue circles indicate SMDs before and after matching, respectively. Dashed vertical lines at ±0.1 represent the commonly accepted threshold for adequate balance. Covariates with SMDs within this range after matching are considered well balanced between groups.
