# Supplementary material for: The Impact of Single-Dose Alirocumab on Efficacy and Safety After Primary Percutaneous Coronary Intervention in Patients With Acute ST-Segment Elevation Myocardial Infarction: A Single-Center Retrospective Real-World Study
Source: Rev Cardiovasc Med. 2026 Mar 12;27(3):47437. doi: 10.31083/RCM47437 (PMC13036518; doi:10.31083/RCM47437)
Supplement: Supplementary file 1 [file 2153-8174-27-3-47437-s1.zip › Supplementary Table 1.pdf]

Supplementary Table 1. Attainment of LDL-C treatment targets at 1 month after PPCI

LDL-C treatment target at 1 month	Alirocumab group	Conventional treatment group
LDL-C < 1.4 mmol/L	20.0%	5.5%
≥50% reduction from baseline LDL-C	14.5%	4.1%
LDL-C < 1.4 mmol/L and ≥50% reduction from baseline	11.6%	1.6%

LDL-C, low-density lipoprotein cholesterol.
