# Supplementary figures and images for: The Impact of Single-Dose Alirocumab on Efficacy and Safety After Primary Percutaneous Coronary Intervention in Patients With Acute ST-Segment Elevation Myocardial Infarction: A Single-Center Retrospective Real-World Study
Source: Rev Cardiovasc Med. 2026 Mar 12;27(3):47437. doi: 10.31083/RCM47437 (PMC13036518; doi:10.31083/RCM47437)

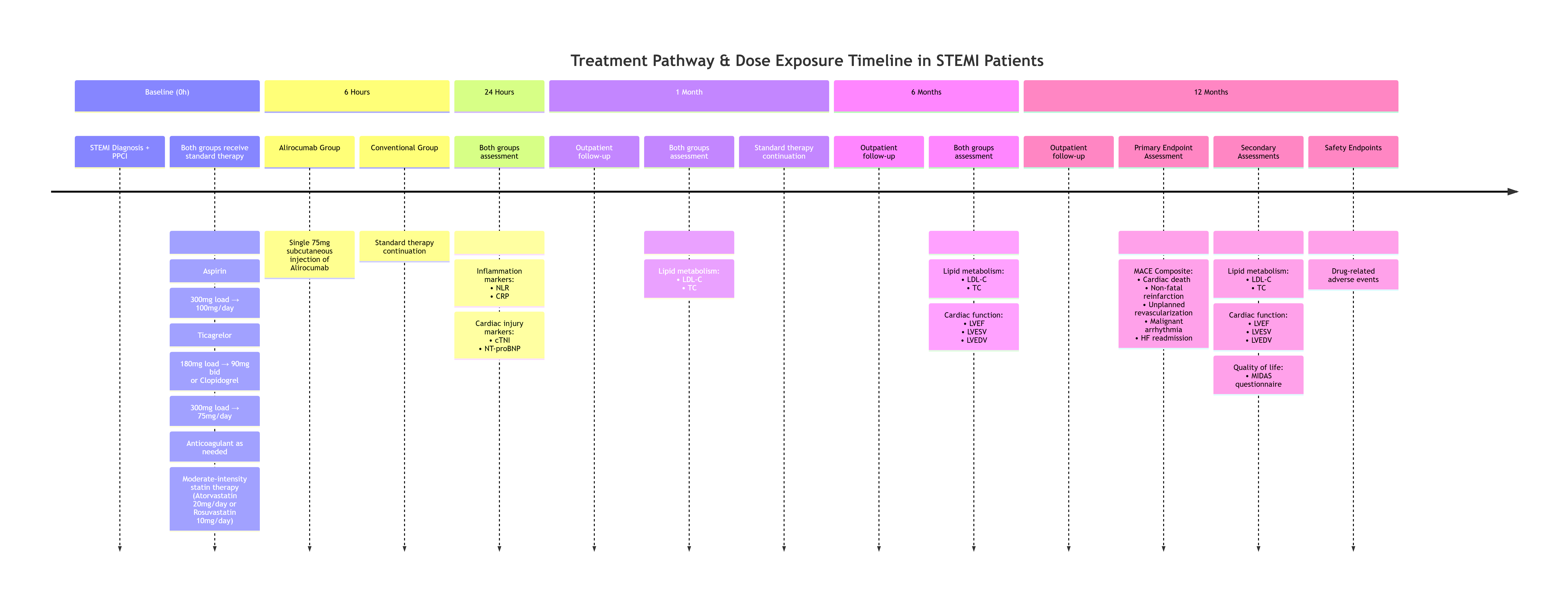

Supplement: Supplementary file 1 [file 2153-8174-27-3-47437-s1.zip › Supplementary Fig. 1.png]

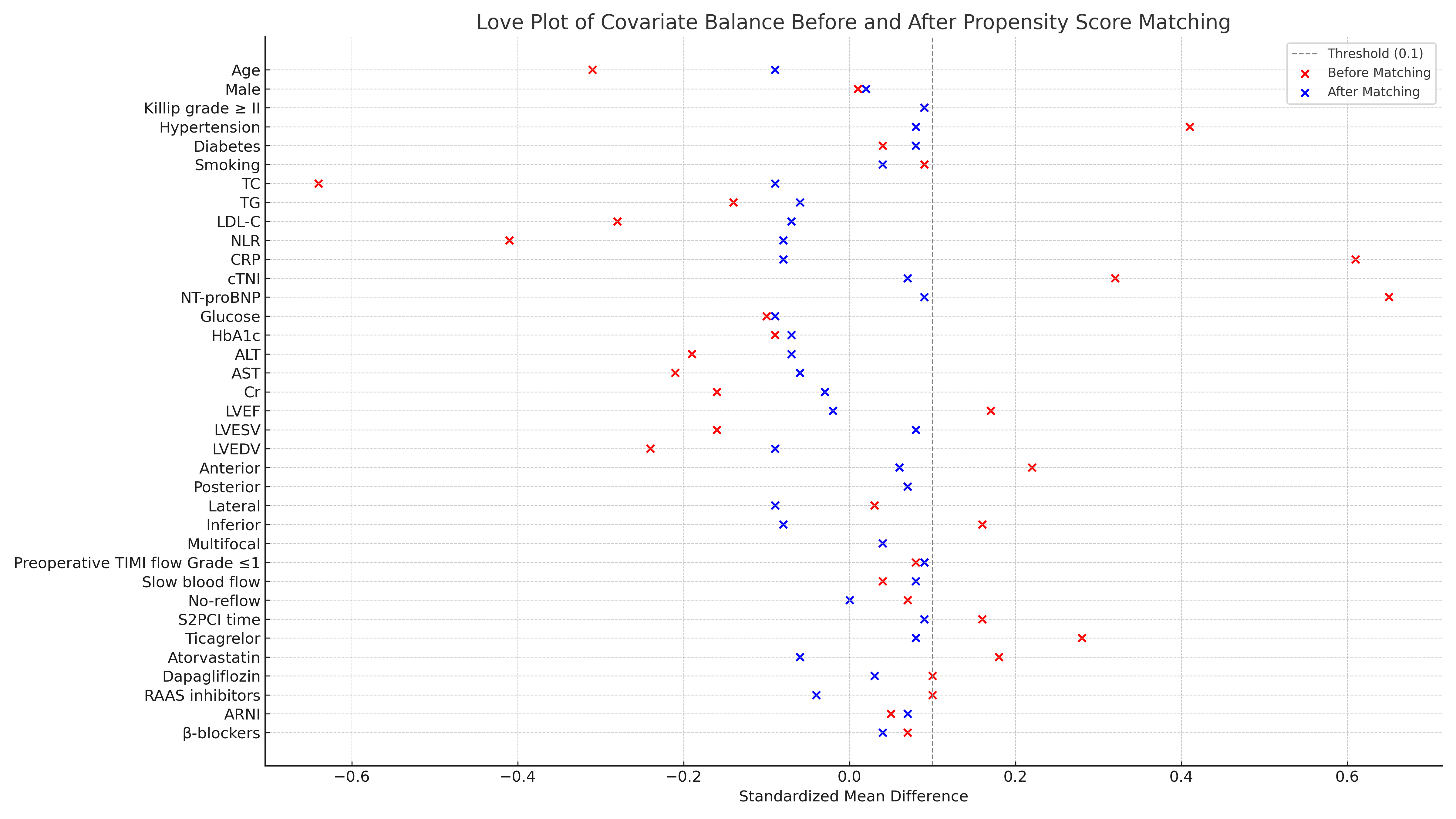

Supplement: Supplementary file 1 [file 2153-8174-27-3-47437-s1.zip › Supplementary Fig. 2.png]
